# Diversity and evolution of *mariner*-like elements in aphid genomes

**DOI:** 10.1186/s12864-017-3856-6

**Published:** 2017-06-29

**Authors:** Maryem Bouallègue, Jonathan Filée, Imen Kharrat, Maha Mezghani-Khemakhem, Jacques-Deric Rouault, Mohamed Makni, Pierre Capy

**Affiliations:** 1 Laboratoire Evolution, Génomes, Comportement, Ecologie CNRS, Université Paris-Sud, IRD, Université Paris-Saclay, 1 avenue de la Terrasse, 91198 Gif-sur-Yvette Cedex, France; 2 Faculté des Sciences de Tunis, UR11ES10 Génomique des Insectes Ravageurs de Cultures, Université de Tunis El Manar, 1002 Tunis, Tunisie

**Keywords:** Aphids, Comparative genomics, *Tc1-mariner*, Transposable elements, MITEs, Molecular evolution

## Abstract

**Background:**

Although transposons have been identified in almost all organisms, genome-wide information on *mariner* elements in Aphididae remains unknown. Genomes of *Acyrthosiphon pisum*, *Diuraphis noxia* and *Myzus persicae* belonging to the *Macrosiphini* tribe, actually available in databases, have been investigated.

**Results:**

A total of 22 lineages were identified. Classification and phylogenetic analysis indicated that they were subdivided into three monophyletic groups, each of them containing at least one putative complete sequence, and several non-autonomous sublineages corresponding to Miniature Inverted-Repeat Transposable Elements (MITE), probably generated by internal deletions. A high proportion of truncated and dead copies was also detected. The three clusters can be defined from their catalytic site: (i) *mariner* DD34D, including three subgroups of the *irritans* subfamily (*Macrosiphinimar*, *Batmar*-like elements and *Dnomar*-like elements); (ii) *rosa* DD41D, found in *A. pisum* and *D. noxia*; (iii) a new clade which differs from *rosa* through long TIRs and thus designated *LTIR*-like elements. Based on its catalytic domain, this new clade is subdivided into DD40D and DD41D subgroups. Compared to other *Tc1/mariner* superfamily sequences, *rosa* DD41D and *LTIR* DD40-41D seem more related to *maT* DD37D family.

**Conclusion:**

Overall, our results reveal three clades belonging to the *irritans* subfamily, *rosa* and new *LTIR*-like elements. Data on structure and specific distribution of these transposable elements in the *Macrosiphini* tribe contribute to the understanding of their evolutionary history and to that of their hosts.

**Electronic supplementary material:**

The online version of this article (doi:10.1186/s12864-017-3856-6) contains supplementary material, which is available to authorized users.

## Background

Genomes contain diverse repetitive DNA sequences of transposable elements (TEs), contributing to their plasticity, adaptability and evolution [1–3]. *Class II* TEs use a “cut and paste” mechanism. They are either autonomous transposons encoding their own transposase or non-autonomous transposons including truncated copies (i.e. copies with only one or no extremity) or copies with internal deletions, but with two intact extremities. Although not encoding for a functional transposase, these shorter copies or miniature inverted repeat transposable elements (MITEs) can be *trans*-mobilized and may reach high copy number with a size homogeneity that distinguishes them from other non-autonomous elements [4].

The *Tc1/mariner* superfamily is ubiquitous and forms the largest group of eukaryotic *Class II* TEs [5]. Its members share several common characteristics and synapomorphies. In particular, the target insertion site is TA, the ORF of autonomous copies encodes a transposase of 282 to 350 amino-acid residues [6]; the transposase contains two helix–turn-helix (HTH) motifs in DNA binding domains and a catalytic triad DDE/D motif [5, 7].

Despite these similarities, two major differences can separate families of *Tc1/mariner*: (i) their complete length from 1 to 5 kb due to their TIR (i.e. the *mariner-*like element *MLE* 13–34 bp long, the *Tc1*-like element *TLE* ranging from 20 to 600 bp), (ii) the DDE/D signature motif in their catalytic domains which corresponds to DD34D for *mariner*, DD34E for *Tc1*, DD37D for *maT*, DD37E, DD39D, and DD41D for *rosa* [8–10].

The *mariner* family, initially described in *Drosophila mauritiana* [11], is one of the best known elements belonging to this superfamily. This element is characterized by a patchy and large distribution among metazoans [12–14], which can be explained, in part, by horizontal transfer (HT), corresponding to its ability to transpose between genomes [15–17]. Due to the great diversity of this family, these elements are classified into several subfamilies based on phylogenetic studies. Five major distinct subfamilies including *irritans, mauritiana, cecropia, mellifera/capitata,* and *elegans/briggsae* were reported [12]. However, 16 minor subfamilies also exist with a more limited distribution [18–20]. Otherwise, the *rosa* monophyletic group, first identified in *Ceratitis rosa* and other Tephritid flies, is closely related to the *mariner* subfamilies [9, 16]. Its main characteristic is a transposase with a DD41D motif, and the nucleotide identity between *MLE* subfamilies is about 40 to 56% [12, 21].

While *MLE* is characterized by a high proportion of inactive copies due to independent accumulation of substitution and indels, known as vertical inactivation [22], three elements, namely *mos1,* found in the fruit fly *Drosophila mauritiana* (*mauritiana* subfamily)*, Famar1* discovered in the common earwig *Forficula auricularia* (*mellifera* subfamily) and *Mboumar9* isolated from the ant *Messor bouvieri* (*mauritiana* subfamily) are still naturally active, and thus able to be mobilized [12, 23–27]. Furthermore, the *Himar1* element from the horn fly *Haematobia irritans* (*irritans* subfamily) has been reconstructed by *in vitro* mutagenesis to restore a potential activity [28, 29]. Due to their wide distribution and ability to successfully invade new genomes by horizontal transmission, naturally and artificially active *mariner* transposons are used as powerful molecular tools in transgenesis and insertional mutagenesis, *inter alia* leading to genetic control strategies of pests [29–32].

In aphid species, only a few studies have described the presence of *mariner* elements. For instance, (i) internal partial sequences of *irritans* and *mellifera* subfamilies were identified *in vitro* by a Polymerase Chain Reaction (PCR) amplification in the soybean aphid *Aphis glycines* [33], (ii) deleted sequences of *mauritiana* subfamily were characterized in seven fruit tree aphid species [34], (iii) in the first version of pea aphid *Acyrthosiphon pisum* genome [35], only three complete sequences, namely *Mariner-Ap_1, 2* and *3,* were published in RepBase [36]. However these sequences shared catalytic motif DD34E and should be more related to *Tc1*-elements.

Nowadays, three aphid’s genomes are available in public databases. Indeed, the recent sequencing of the Russian wheat aphid *Diuraphis noxia* genome (Dnoxia_1.0 reference annotation release 101, http://www.ncbi.nlm.ih.gov) [37], the green peach aphid *Myzus persicae* genome (AphidBase, http://tools.genouest.org/tools/myzus/), and the new annotation of *A. pisum* genome (Acyr_2.0, new reference Annotation Release 102, http://www.ncbi.nlm.nih.gov/) offer an opportunity to investigate the diversity of the *mariner* family within and between aphid species, along with the evolutionary history and dynamics of these elements.

These species belong to the Macrosiphini tribe of the Aphididae family and diverged approximately 42.5 Mya [38]. They are found on different host plants: while *M. persicae* is generalist and found on peach trees or Solanaceae, *A. pisum* and *D. noxia* are specialist, infesting Fabaceae and cereals, respectively. In this paper, we explored these three genomes in order to identify *mariner*-related transposons and their non-autonomous derivatives through a homology-based method using as queries a panel of transposases from databases. Eleven TE clusters from *A. pisum*, seven from *D. noxia* and four from *M. persicae* have been detected. Classification and phylogenetic analysis suggested (i) that these lineages are divided into three groups: the *irritans* subfamily DD34D, *rosa* DD41D and a new group DD40/41D close to *rosa* and characterized by a long TIR, (ii) an evidence of vertical transfer with stochastic losses and several putative HT events. All these data provide new informations about the evolutionary history of these transposable elements in aphids.

## Methods

### Supporting data

The genome of *Acyrthosiphon pisum* and *Diuraphis noxia* are available at NCBI (http://www.ncbi.nlm.nih.gov/). The first contains 541 Mb covering 23,925 scaffolds and the second includes 393 Mb covering 5641 scaffolds [35, 37]. The genome of *Myzus persicae*, presenting 398 Mb and spanning 34,598 scaffolds, is published in aphidbase (The International Aphid Genomics Consortium http://tools.genouest.org/tools/myzus/).

### Data mining

A panel of 18 transposases sequences belonging to the five major *mariner* subfamilies DD34D and to the *rosa* DD41D group (Additional file 1) were used as queries in tBLASTN searches on the three aphid genomes, with default parameters. In order to determine the full sequence of each copy, the best hits were extracted with 5 kb flanking sequences and were manually investigated for TIR searches. Each new complete sequence was then used to retrieve more elements. Truncated copies located at the end of scaffolds and sequences less than 250 bp were further discarded. The sequences closer to DDxE catalytic motif were excluded after a BLASTX search against transposases from this family. Finally, 115 sequences from *A. pisum*, 45 from *D. noxia* and 23 from *M. persicae* were obtained and used in this work.

### Sequence analyses

The nucleotide sequence analyses, including alignment, were done with the Aliview 1.18 [39]. USEARCH6.0 [40] was performed to cluster repetitive sequences using a threshold of 75% identity. Shorter copies flanked by two TIRs and with evidence of transposition (at least 2 copies) were considered as MITEs [4, 41]. Consensus sequences were derived using the relative majority rule.

The putative amino acid sequences were deduced by ExpasyTool (http://web.expasy.org/translate/) and then manually optimized. The nuclear localization sequence (NLS) and the helix-turn-helix (HTH) domain were searched using PSORTII [42] and GYM2.0 [43, 44], respectively (Additional file 2).

### Mining of available eukaryote genomes

The complete nucleotide sequences previously identified were used in BLASTn searches against the nr (non-redundant nucleotide) and WGS (whole genome sequence) databases available on the NCBI. Sequences with more than 60% of nucleotide identity over more than 65% of the length of the query were extracted. These thresholds have been chosen to avoid recovering small fragments and sequences phylogenetically far from the subfamilies here considered. Cases of potential horizontal transfers between aphids and other taxa are considered when elements present more than 90% of identity covering more than 90% of the query sequences as proposed by several authors [17, 20].

### Classification and phylogenetic analysis

The classification is based on the Unweighted Pair Group Method with Variation of Metric UPGM-VM [19], an ascending hierarchical classification analogous to the UPGMA method, with two main differences: (i) there is no arithmetical mean, the nucleotide sequences are aligned by pairs, (ii) the metric varies with the ascending classification and gap is considered as a fifth nucleotide. This variation allows a complete sequence to be gathered in the same group with the corresponding truncated and/or deleted sequences such as MITEs. Thus, the 183 elements extracted from aphid genomes were added to a set of 96 already known complete sequences from the *Tc1-mariner-IS630* superfamily published in GenBank and to 50 sequences found in eukaryote genomes (Additional file 3). MITE classification is based on identity of TIRs, internal sequences of complete transposable elements and on the breakpoints of deletions.

For phylogenetic analysis, the amino acid sequences were aligned with Aliview1.18 [39] and the best-fitting ML model (AIC, matrix WAG + F + I + G) was selected using Protest 2.4 server [45]. Then, the phylogenetic analysis of transposases was computed using MEGA6 [46] with 1000 bootstrap replicates.

## Results

### Distribution and diversity of *mariner* and *rosa* elements within the Macrosiphini tribe

Search of sequences belonging to the main *mariner* subfamilies DD34D and to the *rosa* DD41D group was based on a homology approach (tBLASTN) using a set of 18 known transposases as queries (Additional file 1). We found a total of 115 copies from *A. pisum* clustered in 11 lineages, 45 from *D. noxia* clustered in seven lineages and 23 copies from *M. persicae* distributed in four lineages. A lineage corresponds to a group of sequences that is more than 75% similar and to clear phylogenetic clades (see below).

While 183 copies were extracted, 23 complete and potential autonomous sequences, representing 12.57% of all copies, have been identified in aphid genomes. A low copy number, ranging from one to six, per lineage and per species is observed. More precisely, only ten sequences distributed into nine lineages are found in *A. pisum* genome. All these sequences are named *Apismar.* For *D. noxia*, seven complete copies (*Dnomar*) are grouped into five lineages and only six copies from *M. persicae* (*Mpmar*) are gathered in the same group.

For most of these clusters (14 out of 15), the terminal inverted repeats (TIRs) necessary for transposition have been identified, as well as the TA target site duplication (TSD). The *Apismar4.2* does not display a TSD. Interestingly, the whole nucleotide sequences appear heterogeneous in length. Some clusters with a short TIR (15-32 bp) have a full length of approximately 1.3 kb (i.e. *Apismar1.2, Apismar4.1*), while others (i.e. *Apismar5.1*, *Apismar5.2*) showed sizes longer than 2 kb due to long TIR sequences about 460 bp (Table 1).

**Table 1:** Characteristics of 15 lineages corresponding to complete elements*.* The copy number, clade, length of the element, TIR and ORF, as well as the presence of potentially active copies, are specifically indicated for each complete sequence. The number of copies not truncated by “N” is mentioned in the fifth column. Potentially active copy = existence of at least copy with a complete ORF, with no frameshift or codon stop. In TIR sequences, the mirror sites are mentioned in bold

Clade	Tribe	Species	Lineage name	Complete copy number	Length (bp)	TIR		ORF Length (aa)	Potentially active copy
						Length (bp)	Sequences		
*irritans* DD34D	Macrosiphinimar	*A. pisum*	Apismar1.1	1	1334	28	CGAGGCGTGTCCAGAAAGTAAGTGTACT	354	Yes
			Apismar1.2	1	1317	15	TTCGAAAAGTAAGGG	355	No
		*D. noxia*	Dnomar1.1	1	1347	28	CGAGGCGTGTCCAGAAAGTAAGTGTACT	354	No
			Dnomar1.2	1	1300	20	TWCGAAAAKTAAGGGCCGTT	347	No
		*M. persicae*	Mpmar1.1	6	1334	28	CGAGGCGTGTCCAGAAAGTAAGTGTACT	355	Yes
	*Batmar*-like	*A. pisum*	Apismar2.1	1	1323	30	CGAGGTATGA**CAATAAAATAAY**GAGACTTT	354	Yes
			Apismar2.2	2	1280	22	AAYACCCAGACAAMAWKTATTA	354	No
		*D. noxia*	Dnomar2.1	2	1326	27	YGAKGTGWS**AMATAAAATAAA**CGAGAC	357	No
			Dnomar2.2	2	1344	24	CSWGGTGTGTTCAAAAAGWACYCG	339	No
	*Dnomar*-like	*D. noxia*	Dnomar3.1	1	1360	26	CGAGGGCGGGCTGATAAGTAATGCCT	362	No
*rosa* DD41D	*Crmar2*-like	*A. pisum*	Apismar4.1	1	1355	32	AAGGGTGTCTCAAAAAGAACGCCGGATTTRAA	361	Yes
			Apismar4.2	1	1299	32	GGGTTTTTCAATARRAGCGCTCGAWSTTTSAT	361	No
			Apismar4.3	1	1316	27	GGTGCGGCAGAGCCRACTGACGAGTTT	362	Yes
*LTIR*	DD41D	*A. pisum*	Apismar5.1	1	2307	466	TCACCAATTTAGGGAACACTGAATTTCTCGGCT	370	Yes
	DD40D	*A. pisum*	Apismar5.2	1	2423	460	AATGTGTCAAACTTCTAGAGGTGTTTCTACACC	351	No

**Fig. 1:**
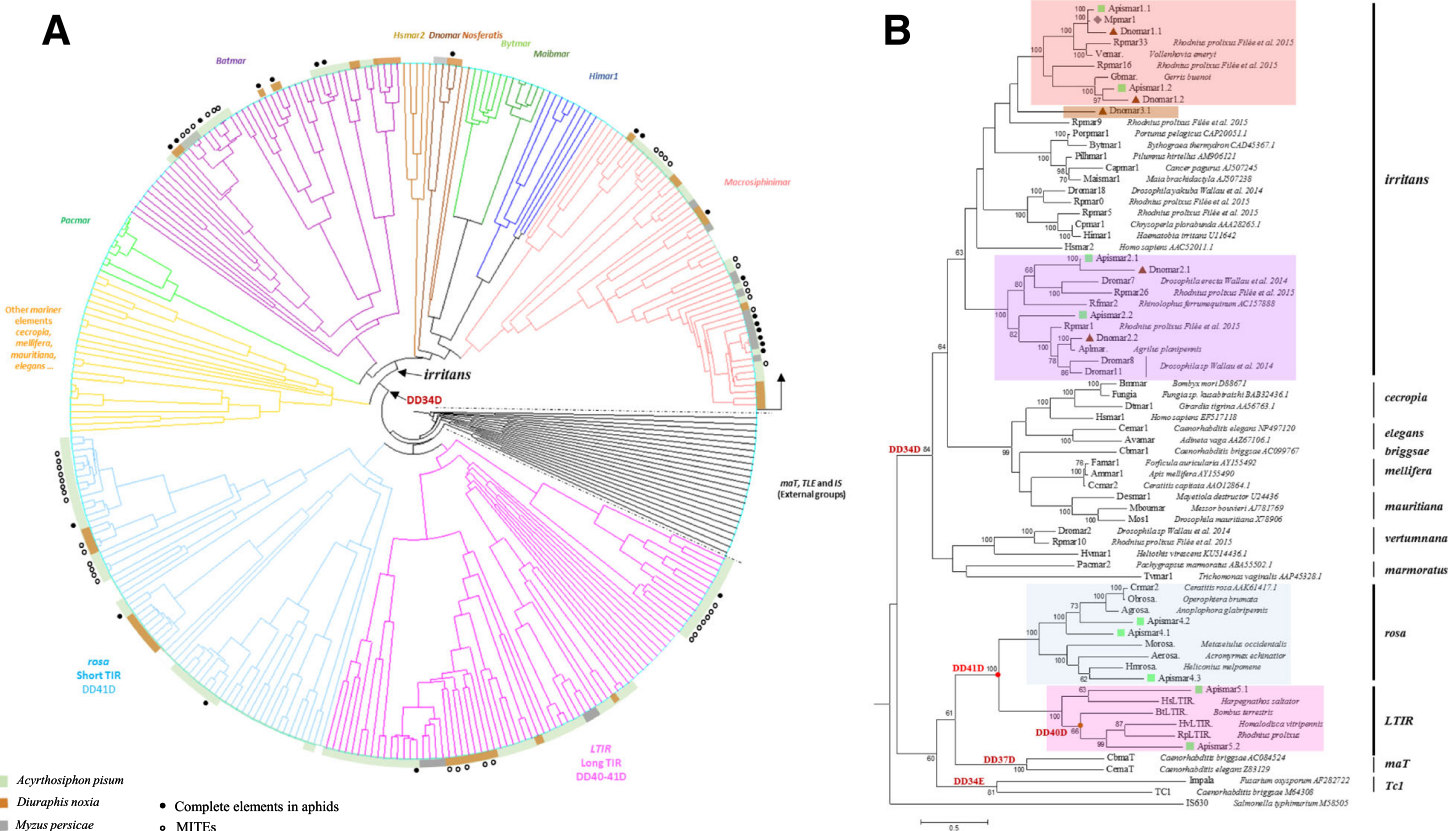
Classification and phylogenetic tree of sequences identified in *Macrosiphini* tribe. **a** Classification of the 115 sequences obtained from *Acyrthosiphon pisum*, 45 from *Diuraphis noxia* and 23 from *Myzus persicae*. These 183 sequences, along with 146 elements belonging to the *Tc1/mariner* superfamily were classified using the UPGM-VM method [19]. References and positions of all these sequences are given in Additional file 3 according to the reading sense indicated by the arrow in the circular tree. Sequences found in *A. pisum*, *D. noxia* and *M. persicae* are given in colour, in green, brown and grey, respectively. Complete sequences are marked by a full black circle and MITEs by an empty circle. **b** The phylogeny based on amino-acid sequences of the 15 lineages. After a search of the best evolutionary scenario (ProTest 2.4), this tree was generated in MEGA6 with the Maximum likelihood (ML) method, using the WAG + F + I + G model. Only bootstrapping values (1000 replications) higher than 60% are written on the branch. Families and subfamilies are indicated in the right-hand part of the tree. The colored rectangles correspond to the different tribes as in A. Green squares, grey lozenges and brown triangles refer to the aphid species *A. pisum*, *D. noxia* and *M. persicae*, respectively. The designation of unpublished sequences extracted from other species than those of the three aphids includes a point (i.e. *Vemar.* from *Vollenhovia emeryi*). Sequences name: *Apismar*: elements from *Acyrthosiphon pisum*, *Dnomar:* elements from *Diuraphis noxia* and *Mpmar*: elements from *Myzus persicae*

Classification of the 183 aphid sequences, based on the 146 nucleotide sequences of the *Tc1/mariner* superfamily, was performed using a UPGM-VM method. This allows all sequences to be dealt with whatever their length, including the distantly related *Tc1* and *Tc3* sequences of animals, plants, fungi and bacteria like *IS630* (Fig. 1, Additional file 3).

Results reveal that 75 copies (18 complete elements and 57 deleted/truncated sequences) belong to the *irritans* subfamily. They can be subdivided into three tribes: the first is widespread in aphids, namely *Macrosiphinimar* (*Apismar1*, *Dnomar1* and *Mpmar1*). The second is close to known *Batmar-*like elements found in the bat *Rhinolophus ferrumequinum* genome. This group includes complete (*Apismar2* and *Dnomar2*) and shorter sequences (deleted or truncated) from the three aphids species. The last tribe, namely *Dnomar*-like element, contains a complete copy from *D. noxia* (*Dnomar3*) and deleted/truncated sequences from *D. noxia* and *M. persicae*.

Furthermore, two other groups can be identified: *rosa* DD41D and a new one close to the latter (Fig. 1, Additional file 3). *rosa* DD41D is represented by 44 copies restricted to *A. pisum* (*Apismar4*) and *D. noxia* genomes. They are clustered with *Crmar2* found in the Diptera Mediterranean fruit fly *Ceratitis rosa.* The second group, characterized by a long TIR, named *LTIR*-like elements, mainly comprises sequences from the pea aphid (*Apismar5.1*, *Apismar5.2*) and may correspond to a new subfamily.

In the same genome, at least four lineages can coexist. However, large differences are observed among species (Fig. 1). Indeed, in *M. persicae*, a potential autonomous element (*Mpmar1*) from *Macrosiphinimar,* related to short sequences, is identified. No *rosa* elements are detected and only deleted/truncated copies belonging to *LTIR*-like, *Dnomar*-like and *Batmar*-like elements are detected. In *D. noxia*, five *irritans* lineages are found. They include potential autonomous elements (*Dnomar*) and a few deleted/truncated copies of the same lineage. Two lineages are composed by short sequences belonging to *rosa* and *LTIR* clades. Furthermore, the genome of *A. pisum* is free of *Dnomar*-like elements. The other lineages are mainly represented by deleted/truncated copies and only a few complete sequences (*Apismar1–5*) can be detected. Hence, the large diversity of these elements among species may reflect the independent evolutionary history of these lineages or specific properties of the genome.

TIRs show a higher degree of identity in the *irritans* subfamily, suggesting a possible recent common ancestor, while they seem to be less conserved in *rosa* and *LTIR* elements (Additional file 4). In addition, TIRs do not present palindromic motifs, but only mirror repeats can be detected in *Apismar2.1* and *Dnomar2.1* belonging to *Batmar*- like elements (Table 1).

Otherwise, the screening of NCBI-nr and WGS databases (Eukaryotes) with the complete elements identified in aphid’s genomes reveals only one sequence having a level of similarity above 90%, with cover queries up to 90%. In fact, it concerns a complete element belonging to the *irritans* subfamily found in the genome of the Coleoptera *Agrilus planipennis,* which is closely related to *Dnomar2.2* from *D. noxia* with 92% of similarity (Fig. 1, Additional file 3).

### Protein and phylogenetic analyses

**Fig. 2:**
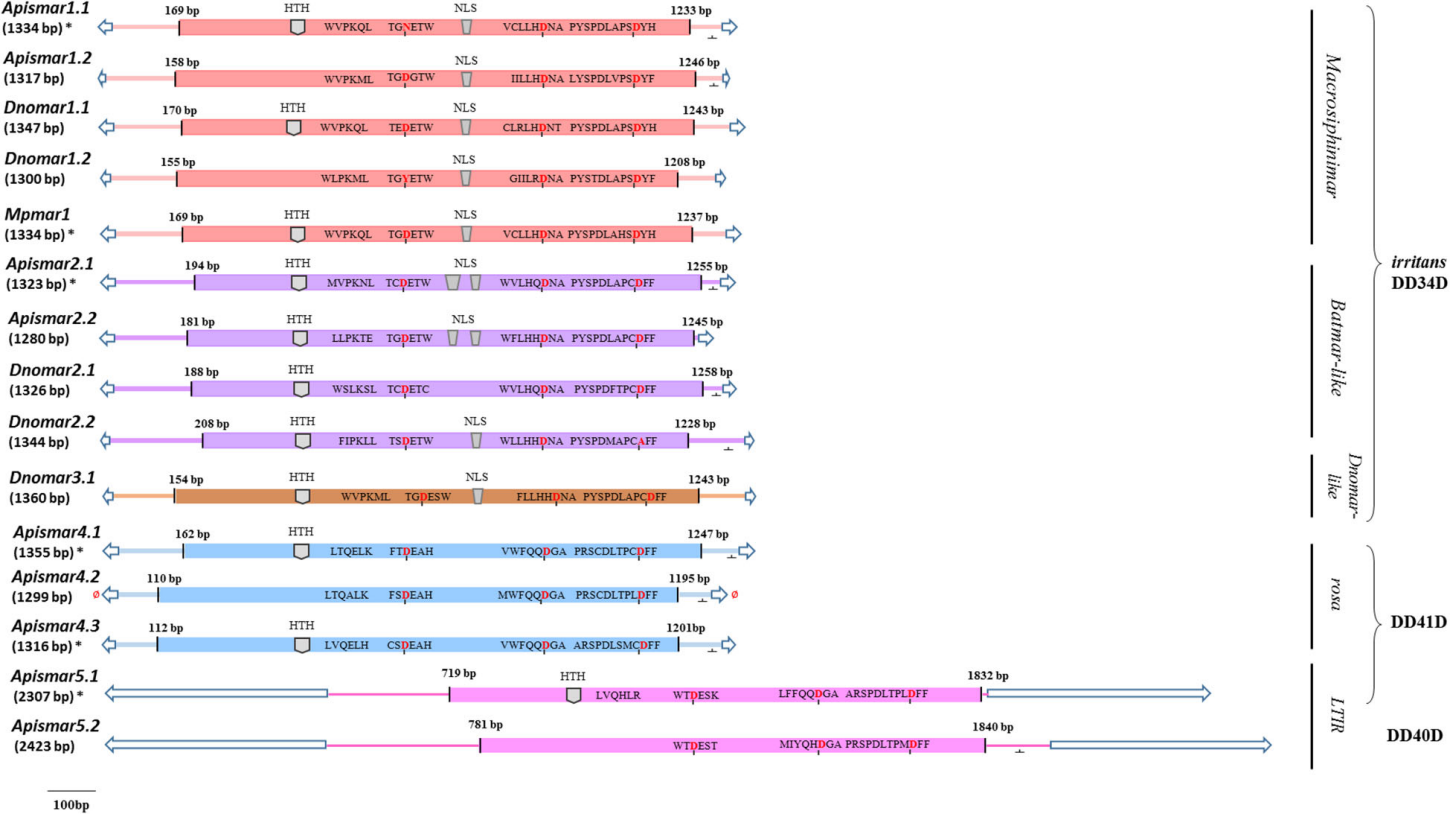
Schematic representation of the 15 lineages corresponding to complete sequences found in aphid’s genomes. The elements are arranged and colored (as in Figure 1) according to the clades they belong to. Potentially active copies are marked with asterisks. The lack of TA (TSD) is marked by a slashed zero in red. Blue arrows indicate TIR, while bold lines represent UTRs. A turned T shows the presence of polyAdenylation site “AATAAA”. In transposase gene, the three catalytic residues containing aspartic amino acids marked in red are indicated. The helix turn helix (HTH) region, the nuclear localization signal (NLS), and motifs related to WVPHEL are also mentioned. Sequences name: *Apismar*: elements from *Acyrthosiphon pisum*, *Dnomar:* elements from *Diuraphis noxia* and *Mpmar*: elements from *Myzus persicae*

The protein sequences of the 15 full clusters are characterized by an ORF encoding about 339 to 370 aa (Fig. 2, Table 1 and Additional file 2). They are aligned with 56 other copies of the *Tc1-mariner* superfamily belonging to non-aphids species. The topology of the ML phylogenetic tree is roughly similar to the classification based on nucleotide sequences (Fig. 1, Additional file 3). Indeed, the five tribes, previously described, are supported by high bootstrap values (98–100%). The percentage of identity between these clades varies from 28 to 59% (Additional file 5).

Only six complete sequences (*Apismar1.1*, *Mpmar1*, *Apismar2.1*, *Apismar4.1*, *Apismar4.3* and *Apismar5.1*) present an intact ORF with no frameshift or codon stop, suggesting that they might be active (Table 1, Additional file 2). An analysis of the transcriptomes of the two species (*A. pisum* and *M. persicae*) was performed using these 6 sequences with a complete ORF. Five sequences (*Apismar1.1, Mpmar1.1, Apismar4.1, Apismar4.3* and *Apismar5.1*) present a full-length transcript, while the last one *Apismar2.1* presents an internal deletion leading to the loss of 140 aa. The sequences related to the conserved motifs, especially WVPHEL and YSPDLA, as well as the catalytic site DD34D considered as the *mariner* signature [47, 48], are detected in most of the sequences belonging to the *irritans* subfamily: *Macrosiphinimar*, *Batmar*-like elements and *Dnomar*-like elements (Fig. 2, Additional file 2). The less conserved motif is WVPHEL, localized between the HTH motif and the first D. The catalytic site is relatively well conserved (7 out of 10) with a length polymorphism between the three residues. Two sequences are deprived of HTH and one of NLS. These three copies are probably inactive.

In the *rosa* clade, close to *Crmar2*-like elements, the catalytic domain is DD41D rather than the canonical DD34D (Fig. 2, Additional file 2). While the NLS motif is lacking, the HTH is located from position 88 to 110 in *Apismar4.1* and from 90 to 112 in *Apismar4.3*.

**Fig. 3:**
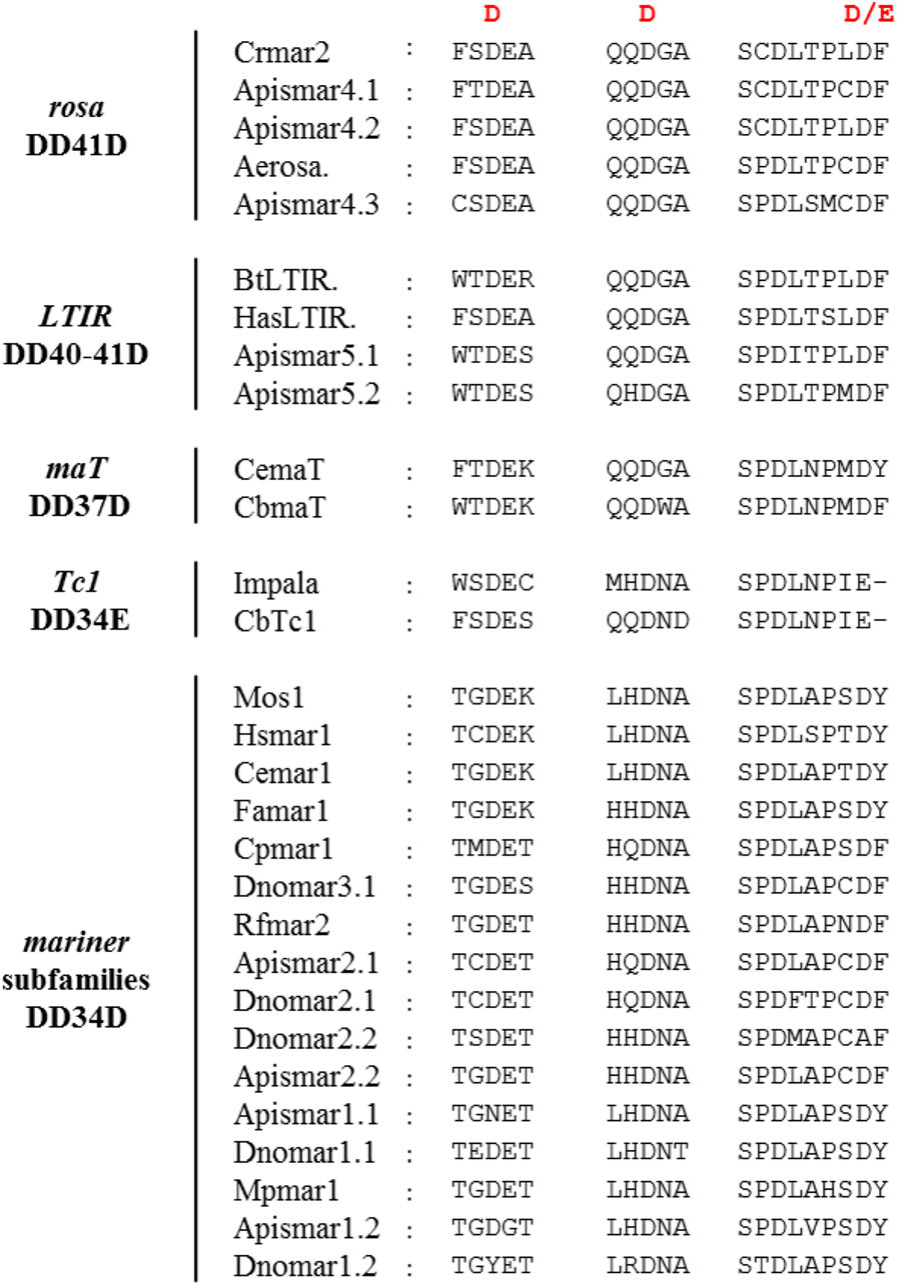
Multiple alignments of catalytic motifs of *Tc1, mariner, maT* families with the 15 lineages identified in aphids

The classification and phylogenetic tree showed the presence of a monophyletic clade related to *rosa* DD41D (43% ± 0.016 of similarity), designated *LTIR*. This monophyletic group, characterized by long sequences (> 2.3 kb) with a long TIR (> 460 bp), can be divided into two tribes based on the transposase similarities. The NLS motif is absent and in the catalytic domain the distance between the second and the third D is of 40 aa for *Apismar5.2* and 41 aa for *Apismar5.1*. Otherwise, HTH motif is only present in *LTIR* DD41D (Fig. 2, Additional file 2). The phylogenetic tree also indicated that *rosa* DD41D and *LTIR* DD40-41D elements are closer to *maT* and *Tc1* than to *mariner* subfamilies (Fig. 1). The comparison of the sequences surrounding the catalytic site is summarized in Fig. 3. The flanking sequences of the second D is clearly distinct between the different groups (*rosa/LTIR/maT* vs the *mariner* subfamilies).

### MITEs occurrence: Structure and evolution

MITEs are defined as short non-autonomous copies which are known to derive from autonomous ones. They do not encode functional transposase but can be *trans*-mobilized thank to the transposase of complete copies.

**Fig. 4:**
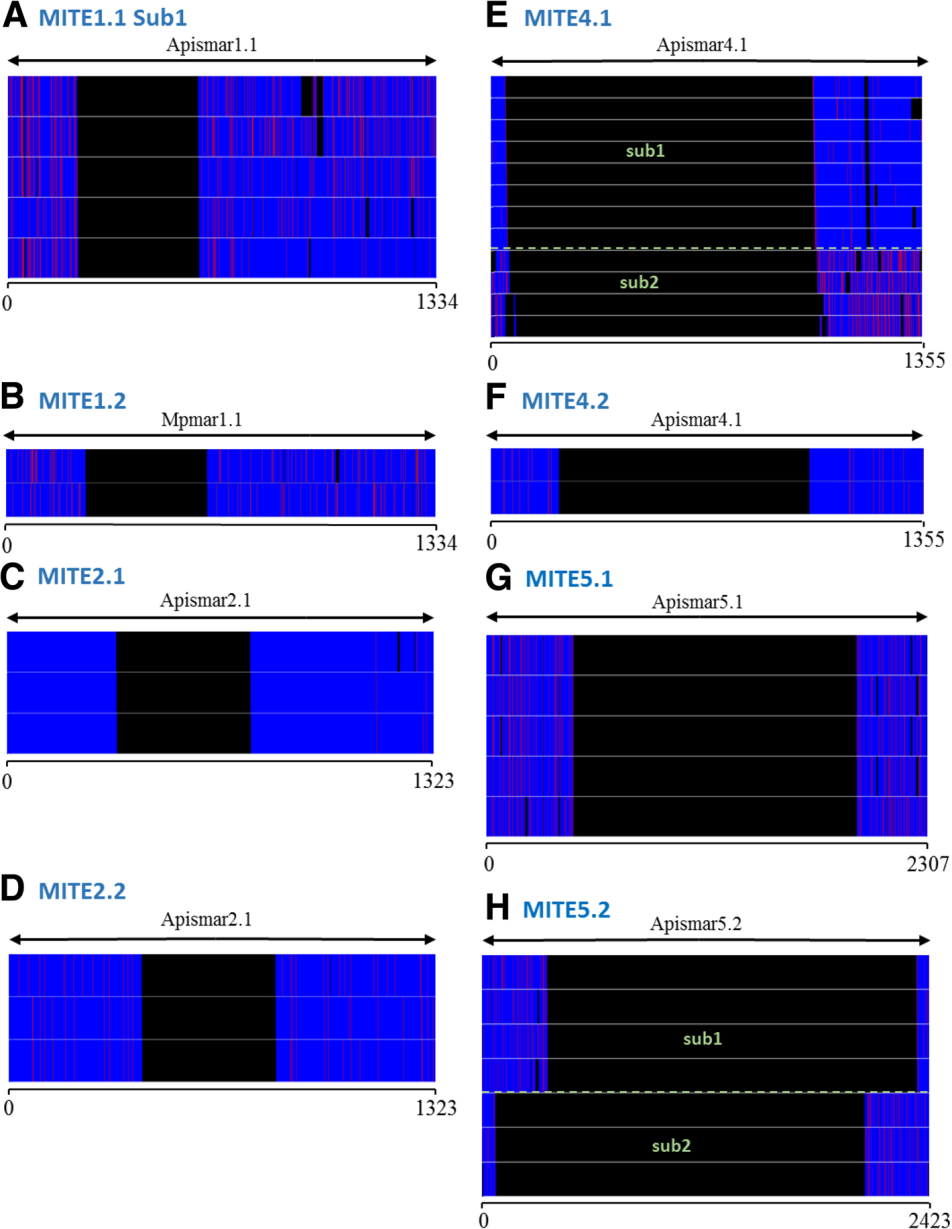
Sequence alignments of MITE lineages with a longer autonomous partner. For each alignment (**a**-**h**), sequences are in blue, showing substitutions in red and gaps in black. The autonomous copies related to MITE and the global structure of the copies are shown on top, with arrowheads corresponding to TIR. Similar copies in length and sequence-defined sublineages (numbered in green). Given the lack of homology with the full potential element, *MITE1.1 sub2* is not represented. **a, c, e** and **h** are found in *A. pisum*, **b** and **d** in *M. persicae*, **f **and **g** in *D. noxia*

MITEs, detected in the present work, represent 43 copies i.e. 23.5% of all extracted sequences. Only the *Dnomar*-like tribe is free of MITEs (Table 2). For the others, there is a large-size polymorphism, and MITEs are clustered into 11 sublineages based on the breaking points of the main internal deletion and the TIR sequences. All of these sequences, except one (*MITE1.1 sub2*), can be related to a full-length copy (Figs. 1 and 4 and Additional file 3). Microhomologies have been found at the breaking points of the internal deletions for most of the MITEs. According to the nomenclature proposed by Negoua et al. [49], they are of the BPEE type for seven sublineages of MITE, and of the BPNN type for two other sublineages (Table 2). For the remaining (*MITE1.1*) no microhomology can be detected.

**Table 2:** List of MITEs detected in the aphid’s genomes. NR = no related autonomous copy identified. Presence of short direct repeat (microhomologies) in the region of deletion breakpoints are indicated: BPEE for Breaking Point Exact Exact and BPNN for Breaking Point Near Near (according to the nomenclature proposed by Negoua et al. [49])

Clade	Tribe	Species	ID MITE	Length (bp)	Sublineage	Copy number	TIR sequences	Autonomous element related to MITE		Breakpoints
								sequences	Average identity (%)	
*irritans* DD34D	Macrosiphinimar	*A. pisum*	MITE1.1	923–1165	sub1	5	CGAGGCRTGTCCAGAAAGTAAGTGTACT	Apismar1.1	90.8	-
					sub2	4	CGAGGCGTGTCCCAAAARTAAGGTCTCCAT	NR	-	
		*M. persicae*	MITE1.2	959, 1007	sub1	2	CGAGGCGTGTCCWGAAAGWAAGTGTACT	Mpmar1.1	92	BPEE
	*Batmar*-like	*A. pisum*	MITE2.1	908–931	sub1	3	CGAGGTRTGACAATAAAATAACGAGACTTT	Apismar2.1	98.6	
		*M. persicae*	MITE2.2	908–912	sub1	3			97	BPNN
*rosa* DD41D	*Crmar2*-like	*A. pisum*	MITE4.1	349–548	sub1	8	AAGGGTGTCTCAAAAAGAACGCCGGATTTRAA	Apismar4.1	94.5	BPEE
					sub2	4	RGGRTRYCWCAAAAARAAGSGYGGATTTKRAA		74.6	
		*D. noxia*	MITE4.2	578	sub1	2	WAGGGTGTCTCAAAAAGAACGCCGGATTTRAA		97	
*LTIR*	DD41D	*D. noxia*	MITE5.1	790–822	sub1	5	TCACCAATTTAGGGATCACTGAATTTCTCGGC…	Apismar5.1	86.2	BPEE
	DD40D	*A. pisum*	MITE5.2	411–441	sub1	4	AATGTGTCAAACTTCTAGAGGTGTTTCTACAC…	Apismar5.2	90.25	BPEE
					sub2	3			90	BPNN

In the *irritans* clade, represented by the *Macrosiphinimar* tribes and *Batmar*-like elements, only *A. pisum* and *M. persicae* contain MITEs, with size varying between 908 and 1165 bp. The first tribe (*MITE1.1*) includes nine copies from the pea aphid clustered in two sublineages (*sub1* and *sub2*) which only share the first 12 nucleotides of the TIRs. An additional lineage (*MITE1.2*), closely related to *MITE1.1sub1,* is found in *M. persicae*. These two sublineages present similar TIRs and an average identity of 81.8%. However, they do not have similar breaking points (Fig. 4). These two types of MITEs are related to putative autonomous copies found in each species (*Apismar1.1* and *Mpmar1.1* respectively) showing 99% of identity.

A similar situation is observed for the *rosa* clade when *MITE4.1sub1* and *MITE4.2* are compared. The *MITE4.1* lineage, includes 12 copies with lengths from 349 to 548 bp, comprised two sublineages. Although clearly related, these sublineages seemed to result from independent internal deletions of the *Apismar4.1* complete element. The *D. noxia* genome contains two copies of a MITE of 578 bp (*MITE4.2*) which are also closely related to the autonomous element *Apismar4.1* (Fig. 4).

For the *LTIR* DD41D tribe, *MITE5.1,* only found in *D. noxia,* comprises five copies (790–822 bp) with the same breakpoints, and are related to the autonomous element *Apismar5.1*. No *MITE5.1* was retrieved in the *A. pisum* genome. Furthermore, *MITE5.2* of *LTIR* DD40D tribe identified in the pea aphid is composed of seven short copies (411 and 441 bp). They are divided into two sublineages depending on the breakpoint positions, probably resulting from independent internal deletions (Fig. 4).

Globally, these results show that (i) MITEs in aphid species are less frequent than in *Drosophila ananassae* (about 240 copies) [41] and in *Rhodnius prolixus* (about 400 copies) [20]; (ii) *irritans* clades do not generate MITEs smaller than 900 bp, in contrast to *rosa* and *LTIR*-like elements clades; (iii) three MITE sublineages (*MITE2.2*, *MITE4.2* and *MITE5.1*) are closely related to autonomous copies found in other species; (iv) orphan MITE sublineages can be detected with no full-length partner (*MITE1.1 sub2*). In the later case, it cannot be excluded that active copies still exist in other populations or closely related species.

The distribution of MITEs and their relationship with full-length elements show that their phylogeny is inconsistent with that of the species. Several scenarios involving the existence of ancestral polymorphism, current population polymorphism (presence/absence of autonomous copies and/or MITEs), stochastic loss of autonomous copies and/or horizontal transfers can be proposed.

**Fig. 5:**
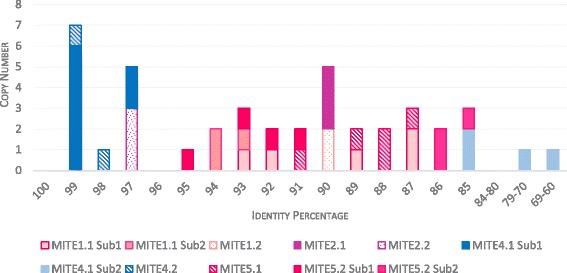
Evolution analysis of different MITEs sublineages. Based on the comparison of consensus with copies, the similarity rates are identified. While copy sublineages with a high level of similarity present recent invasion, the decrease of this percentage refers to an ancient element. Filled, hatched and dotted patterns correspond to *A. pisum*, *D. noxia* and *M. persicae,* respectively. Colors match to the different tribes as in Fig. 1

To infer the dynamics of MITEs identified in the aphid genomes, we generated consensus sequences for each sublineage in order to estimate their period of amplification from their percentage of divergence, as proposed by Le Rouzic et al. [50] and Wallau et al. [41]. Except for two sequences of the *MITE4.1 sub2* showing 69 and 72% of identity with the consensus of this lineage, all others exhibit a level of identity higher than 85% (Fig. 5). While the transposition rate (*trans*-mobilization) of these copies is unknown, we observed that some of them are almost identical (97–99% of identity) suggesting that these copies are still *trans*-mobilizable or were recently inactivated. The remaining sequences (identity level from 85% to 95%) are less conserved and probably correspond to ancient *trans*-mobilization, and are no longer mobilizable.

## Discussion

The three species of aphids, *A. pisum*, *D. noxia* and *M. persicae,* present different genome sizes (541 Mb, 393 Mb and 398 Mb respectively), which correspond to different TE equipment [35, 37], i.e. 38% and 11.5% for the first two species (no information being available for *M. persicae*), suggesting as previously proposed that the contribution of TEs to genome size variation is greater relative to other sources of variation [41, 51, 52].

In the present work, we focused on a survey of *MLE*-related elements in aphid genomes. Our data are in agreement to the previous observation since a total of 115, 45 and 23 sequences, extracted from *A. pisum*, *D. noxia* and *M. persicae*, respectively, are clustered into 22 lineages. The relative abundance of *MLE*-related elements in these three aphids’ genomes is low compared to other insect genomes. For instance, *mariner* subfamilies are represented by 10,836 copies in the 700 Mb genome of the Hemiptera *Rhodnius prolixus* [20] and 642 copies in the 156 Mb genome of the *Drosophila eugracilis* [41]. Otherwise, the *Tc1-mariner* superfamily is poorly represented in each aphid genome compared to other superfamilies of DNA transposons, such as *piggyBac* or *hAT* (personal data). This observation might be an illustration of the competition that may occur between superfamilies as described by Abrusán and Krambeck [53]. However, today without a complete and detailed overview of TE equipment of these genomes, we do not have strong arguments to conclude that such a result is due to competition.

In the *mariner* family, only members of the *irritans* subfamily are identified in the aphid’s genomes. They belong to the *Macrosiphinimar*, *Batmar*-like and *Dnomar*-like tribes, and are characterized by the DD34D catalytic site. Moreover, only three lineages might still be active (*Apismar1.1*, *Mpmar1.1* and *Apismar2.1*). No sequence related to other *mariner* subfamilies (i.e. *mauritiana, mellifera, cecropia, elegans*) is found in these genomes, although they have been identified in vitro in other species belonging to a closely related aphid species such as *Aphis glycines* [33] and seven tree aphids [34].

However, sequences belonging to the *rosa* family (initially closely related to the *mariner* family [9]) have been detected in *A. pisum* and *D. noxia*; and a novel clade (*LTIR*-like) has been identified. Such *LTIR* elements including the DD41D motif, designated as *Lsra* transposons, were described by Zhang et al. [54]. This clade is closely related to the *rosa* subfamily but is characterized by long TIRs (about 460 bp vs 28-32 bp). Moreover, conservation of some specific amino acid residues in their catalytic region, especially the final aspartic acid (D) rather than glutamic acid (E), and phylogenetic analysis revealed that *rosa* and *LTIR*-like elements are more closely related to *maT* elements than to *Tc1* and *mariner* ones. Therefore, we suggest that *rosa* DD41D and *LTIR*-like elements constitute a large new family belonging to *Tc1/mariner.*

Distribution, diversity and phylogeny of these elements in the three aphids’ genomes are probably the result of vertical transmissions associated to an ancestral polymorphism. In such a situation, closely related sequences derived from the same ancestral copy can be found in several species, while copies derived from different ancestral copies and found in the same genome, can be more distantly related (see for instance [55–57]). Host genomes are also able to repress TE activity [58, 59], leading to their elimination by stochastic loss or vertical extinction. Therefore, the absence of members of the *rosa* family may be due to a stochastic loss during the evolutionary trajectory of *M. persicae*. A similar observation was illustrated in some *Drosophila* species for *mariner* subfamilies [41, 60].

The high level of similarity between MITEs and autonomous partner indicates that short sequences are internally deleted elements, deriving from complete copies. Most of them exhibit direct repeat microhomologies exactly (BPE) or nearly (BPN) to the deletion breakpoints, suggesting that these internal deletions are probably due to abortive gap repair [49, 61, 62]. However, MITEs and related complete copies can be found in two different species, as described in the *R. prolixus* and *Drosophila* genus [20, 41]. This is the case for *MITE2.2, MITE4.2 and MITE5.1*. To explain such observations, two scenarios can be proposed. First, the ancestral autonomous element at the origin of MITEs may have been lost after the MITE amplification, but was maintained in another species. Another hypothesis consists in the emergence of MITEs after internal deletion(s) of a complete copy, these MITEs being then mobilized by the transposase of another copy closely related to the first one.

Finally, horizontal transfer may also occur for all these sequences between distantly related species. For instance, the *mariner* autonomous transposon *Dnomar2.2* from *D. noxia* is closely related to the sequence of *Agrilus planipennis*. Despite a divergence time of about 361 Mya between these two species (http://www.timetree.org/home), the phylogenetic tree of these elements is inconsistent with that of the species. Moreover, HT could also explain the patchy distribution of MITE elements in aphids. However, in all these cases, the transfer mechanism(s) remain unknown and only propositions are suggested, like those proposed in Silva et al. [63] and Loreto et al. [64].

## Conclusion

Our results represent the first in silico evidence of diversity and possible evolutionary scenarios of elements belonging to the three clades: *irritans*, *rosa* and a new one named *LTIR*-like elements in aphid genomes. This latter clade is characterized by long TIRs and subdivided into two distinct subgroups based on the catalytic domain signature DD40D or DD41D. Moreover, based on protein and phylogenetic analyses, the *rosa* and *LTIR* transposons are related to *maT* DD37D elements, indicating a recent common ancestor. We also demonstrated the presence of several MITE lineages deriving from internal deletion of autonomous elements. Finally, this study proposes an update of the classification of the *Tc1/mariner* superfamily. Data analyses will offer a basis for future research aiming to understand the role of transposable elements during evolution and to develop biotechnological applications for the genetic control of aphid species.

## Additional files

Additional file 1. *mariner and* rosa transposases sequences used as queries in the tBLASTN search (Species, Clades, Accession number). (PDF 408 kb)

Additional file 2. Amino acid sequences of the transposase of the 15 complete elements. The three aspartic residues of the catalytic domain are marked in red, the sequences related to the WVPHEL-specific motif of the DNA binding domain are indicated in black bold as well as the helix turn helix (HTH) region (underlined) and the NLS (in blue). Stop codons are represented by asterisks (*). (PDF 425 kb)

Additional file 3. Sequences classified by UPGM-VM method according to the reading sense indicated by the arrow in the circular tree. Deleted or truncated sequences are indicated by an asterisk (*). (PDF 276 kb)

Additional file 4. TIR sequences for each clade. TIR consensus per clade was generated using the Web-Logo server (http://weblogo.berkeley.edu/logo.cgi). At each position the nucleotides are stacked one on top of another with the most frequent one on the top. It displays the frequency of bases at each position, with height indicating the proportion of occurrence. The vertical scale is in bits with maximum of two bits possible at each position, indicating that there can be possibility of four different bases at each position. For LTIR- like elements, only the first 57 nucleotides are presented. (PDF 111 kb)

Additional file 5. Pairwise divergence matrix between amino acid lineages. Fifteen complete sequences have been aligned using Aliview, The alignment was then transferred in GENEDOC software [65] to obtain the identity percentage. Sequences name: *Apismar*: elements from *Acyrthosiphon pisum*, *Dnomar:* elements from *Diuraphis noxia* and *Mpmar*: elements from *Myzus persicae*. (PDF 354 kb)

## Abbreviations

BPEE: Breaking point exact exact
BPNN: Breaking point near near
HTH: Helix turn helix
LTIR-like elements: Long terminal inverted repeats like elements
MITEs: Miniature inverted-repeat transposable elements
MLE: *mariner*-like element
NCBI-nr: Non-redundant nucleotide
NLS: Nuclear localization signal
TE: Transposable element
TIR: Terminal inverted repeats
TLE: *Tc1*-like element
TSD: Target site duplication
UPGM-VM: Unweighted pair group method with variation of metric
WGS: Whole genome sequence
