# Supplementary material for: Diversity and evolution of mariner-like elements in aphid genomes
Source: BMC Genomics. 2017 Jun 29;18:494. doi: 10.1186/s12864-017-3856-6 (PMC5490172; doi:10.1186/s12864-017-3856-6)
Supplement: Supplementary file 1 — mariner and rosa transposases sequences used as queries in the tBLASTN search (Species, Clades, Accession number). (PDF 408 kb) [file 12864_2017_3856_MOESM1_ESM.pdf]

**Additional file 1.** ***mariner and rosa*** **transposases sequences used as queries in the tBLASTN search (Species, Clades, Accession number).**

| Transposase | Species | Clades | | Accession number |
|---|---|---|---|---|
| | | Catalytic motif | ID name | |
| Cbmar1 | *Caenorhabditis briggsae* | DD34D | *briggsae* | AC099767 |
| Avamar | *Adineta vaga* | | *elegans* | AAZ67106.1 |
| Cemar1 | *C. elegans* | | | NP497120.1 |
| Gtmar1 | *Girardia tigrina* | | *cecropia* | CAA56763.1 |
| Hsmar1 | *Homo sapiens* | | | U52077 |
| Bytmar | *Bythograea thermydron* | | *irritans* | CAD45367.1 |
| Cpmar1 | *Chrysoperla plorabunda* | | | AAA28265.1 |
| Fungia | *Fungia sp. Kusabiraishi* | | | BAB32436.1 |
| Himar1 | *Haematobia irritans* | | | U11642 |
| Hsmar2 | *H. sapiens* | | | AAC52011.1 |
| Rfmar | *Rhinolophus ferrumequinum* | | | AC157888 |
| Ammar | *Apis mellifera* | | *mellifera* | AY155490 |
| Ccmar2 | *Ceratitis capitata* | | | AAO12864 |
| Famar1 | *Forficula auricularia* | | | AY155492 |
| Desmar1 | *Mayetiola destructor* | | *mauritiana* | U24436 |
| Mboumar | *Messor bouvieri* | | | AJ781769 |
| Mos1 | *Drosophila mauritiana* | | | X78906 |
| Crmar2 | *Ceratitis rosa* | DD41D | *rosa* | AAK61417.1 |
