# Supplementary material for: Diversity and evolution of mariner-like elements in aphid genomes
Source: BMC Genomics. 2017 Jun 29;18:494. doi: 10.1186/s12864-017-3856-6 (PMC5490172; doi:10.1186/s12864-017-3856-6)
Supplement: Supplementary file 2 — Amino acid sequences of the transposase of the 15 complete elements. The three aspartic residues of the catalytic domain are marked in red, the sequences related to the WVPHEL-specific motif of the DNA binding domain are indicated in black bold as well as the helix turn helix (HTH) region (underlined) and the NLS (in blue). Stop codons are represented by asterisks (*). (PDF 425 kb) [file 12864_2017_3856_MOESM2_ESM.pdf]

Additional file 2. Amino acid sequences of the transposase of the 15 complete elements. The three aspartic residues of the catalytic domain are marked in red, the sequences related to the WVPHEL-specific motif of the DNA binding domain are indicated in black bold as well as the helix turn helix (HTH) region (underlined) and the NLS (in blue). Stop codons are represented by asterisks (*).

Apismar1.1

MSIESAAKCEIRAVIRYLVAKEKSPHEIFNEVRTVYGEGHMNRTSVYKWCREFKNDRTNVHDDLRSGRPSILTDD
IVKKSRMRFVDRRLTLDELSAMFPQLSRSLLHETITETLGFHKLCAR**WVPKQL**TEQHMLNRVQASREFLERYELD
GDNFLKSIVTG**N**ETWVAHYTPETKRQSEQWRHTTS**PSTKKFN**TTISAKKIMASVFWDHKGIILIEYLPQGETINA
ARYCETLKKLRRAIQNKRRGLLTSGVCLLH**D**NARPHTANVTKQLLDSFGWDVLNHTPYSPDLAPS**D**YHLFTSLKK
HMGGKKFSADEEVKGAVDKWTKEMAAEFYEAGIKKLICRLTTCIERNGDYVEK

Apismar1.2

MSAIVAAPASCEVRTVIRFLCAKRSSAAEIHGELCLAYGLTVMSEGKIRWCRDFKNGRTNVHDEERSGRPSMQTD
EIVSLVDQKLRFDCRLTISALADEFPNLARTTVYTIITEKLGYHKLYAR**WVPKML**TDQHKEQRIMSSGREFLNRY
RQDGDNLFSHIVTG**D**GTWISYINPETKQQSMQWRHSTS**PKQKKFK**QTPYTSRKMMATVFWDEKSVLLVDFMERGT
TITAQVYCETLNKLRCAIQNRRRGKLSSSIILLH**D**NARPHTAAKTQEKIDFR*ELFNHPLYSPDLVPS**D**YFLFFH
FKKWLVGQRFENDKELNAVENWFNSQAANFYADGLRKLVKRYEKCFEINGNYVEK

Dnomar1.1

MSIESATMCEIRAVIRYLDAKEKSP*GIFNEVRTTYIEGNNINRTSVYKWCREFKNNRTNVHDDLRSGRPSVLTD
DIVKKVENAVCDDRRLTLDELSAIFPQLPRSLIHETITETLGFHKLCAR**WVPKQL**TDQHMLNRVQASREFLERYE
LDGDNFLKSIITE**D**ETWVAHYTLETKRQSEQWHHTTS**PSTKKFK**TIISAKKIMALVFWDHKGIIFIKYLPQGETI
NAARYFETLKKLRRAIQNKKRGLLTSGDCLRLH**D**NTKPHMANVSKQLLDSFGWDVLNHPPYSPDLAPS**D**YHFFT
SLKKLVVGKKFSTDEEVKGAVDKWTKEVFYGAGIKKFICCLITCI*RDGDYAEK

Dnomar1.2

MSTIIPAPTSCEIRAVIRFLCAKRSSAAEIHQELFLVYGPNVMSEGKIRQWCRDLKNGRTNVHSMQTDEIVSLMD
QKPRTNRRLTFSTLADEFPNL*RTIVYTIVTEKLGYHKLCAR**WLPKML**TDQHQE*RITSG*EFLIHYRQDGDNLF
SHIATG**Y**ETWISYINPETKQQSMQWCRSTSSK**PKKFK**PTPCTSRKMIATVFWDEKGVLLVDFMERGTTITAEVYC
ETLNKLRRAIQN*RSGK*SGIILR**D**NARPHTAAKTQEKIQDFRWKLLNHPPYSTDLAPS**D**YFLFLHFKKWLGEQR
FENDKELKNAVENWFKSQNEFYTDELRKLVKWYEKCLEVNGDYVEK

Mpmar1.1

MSIESAAKCEIRAVIRYLVAKEKSPHEIFNEVRTVYGEGHMNRTSVYKWCREFKIGCTNVHDDLRSGRPSILTDD
IVKIVENAVRDDRRLTLDELSAMFPQLSRSLLHETITETLGFHKLCAR**WVPKQL**TEQHMLNRVQASREFLERYEL
DGDNFLKSIVTG**D**ETWVAHYTPETKKQSEQWRHTTS**PSTKKFK**TTISAKKIMASVFWDLKGIILIEYLPQGETIN
AARYCETLKKLRRAIQNKRRGLLTSGVCLLH**D**NARPHTANVTKQLLDSFGWDVLNHPPYSPDLAHS**D**YHLFTSLK
KHMGGKKFSADEEVKGAVDKWTKEMAAEFYEAGIKKLICRLTTCIERNGDYVEK

Apismar2.1

MLDIKIEQRVNIKFLVKLKKTAAESFRMLCEVYGEECLSRARVFEWHKRFCSGREDVEDDDRSERPTTSSTNENV
EKIDKIIRQDRRLSVRAVAEMVNIDRESVRKILVENLNMNKVCAK**MVPKNL**TIDQKFNRKEICSDTLKIIKDDPS
FINNIITC**D**ETWIFTYDPET**KRQSMHWKTPTSPRMKK**ARMSKSKFKAMLIVFFDIKGIIFVEWVPSGQTVNQYYY
KEVLIKLRERV**RKKRP**DLWKNGWVLHQ**D**NAPAHSAFSIQRYLTEKKISVLQHPPYSPDLAPC**D**FFLFPKIKSLLK
GTHFQTVDDVKMKTAELLKGLNESDWQHCFQEWQRRMQQCIDAEGRYFEGDNH

Apismar2.2

MDNITEQRACFKFCSKIGKNATETLELIKLAFGDVSLSRCVTFDWFRRSKEGRISIEDDYRPGRPSSSKTNDTID
LVRNKIRNYRRLTVREVANEVGISIGTCHSILSDELSMKRVSAK**LLPKTE**EQMEHRIEVCLELKNRVSNDPNFIK
SIITG**D**ETWVYGYDPKTKVQSSQWKTANSSR**PKKCRQI**RSNIKAMLIVFFDFFGLVHYEFVPTGQTINQVFYK*V
VLKRLREKVC**RKRP**EVWKSKSWFLHH**D**NAPAHSALSIREFLASKNIPVVPHPPYSPDLAPC**D**FFLFPRLKSTLKG
HRFVDVNETIHNATQELKAITMKEIQRCFKKWQDRWDHCIEAKGHYFEGDPFK

Dnomar2.1

MLGIKFEQR*NIKFFCEIKKKIAVESFHILCEFYGEERLSRVCVFELHKERMSKMMIVLDVLRPTTPSTNENVEK
IDTIIRK*RRLSVRAVTEMVNIDRESVRKILVENLNMKKMCAK**WSLKSL**TVDQKFNRKEICSDTLKIIKDDPSFI
NNIITC**D**ETCIFTYDTETKRQSMHWKTVRHQVSQE*RKHE*TSQNSKQFSLFSLTLRE*LFLEWVPSGQTVYQYY
CKEILIKLKEHIRKK*LNLWKNGWVLHQ**D**NAPAHSAFSIQRFLTEKNIFILQHPPYSPDFTPC**D**FFLFSKIKSLL
KNGTNNFQTVDDVKM*TAELLKGLTESDWQYCFQEWQRRMQQCVDDE*RYFEGDNH

Dnomar2.2

MEELKSQRIFIKFCVKNEIKCSKVCELLQKAYGESAMKKTIVNEWYKRYQDGRKDVEDDKRSGRPSTSIIDANVK
KVEKMVVNDRRITIIREVADEVGISIVSCQNIFSNVLGLKRVAAK**FIPKLL**NFDQKNNRMNVAQELLNDVNVDPS
LLERVITS**D**ETWVYGYEVETKAQSSTWKHSTS**PRAKKARQV**RSNVKVLLTVFFDFNGIVHQEFLPQGRTVNKEYY
LEVMRRLREAIRKKRPDIWKNNSWLLHH**D**NAPAHSSLLVHNFLAKNNTAVMPQPPYSPDMAPCAFFLFPMLKRHM
KGQRFSSIEEIKAESLRVLKDMPKSEYQECFEDWKKSLA

Dnomar3.1

MESIITAPSDVRFKQRAVIEFLVAENVKPVDIHRRLLAVYGNQTLDVSSVRRWALRVKGSEVGKAIITDQDRSGR
PVTVTDEGLVTRPIKQKVDDLVKGNRRIKQSEIAIALGISKERVQHILCELEYRKICTR**WVPKML**TEEMKQNRVE
ICRQLLLRLNVRENFLNIMVTG**D**ESWVHHYGPENKRQSMEFRHKTSPA**PKKFK**VQASAGKVMLTVFWDSKGVIHT
EYLEKGSTINSIRYIEALKKLKKRIKRVRPNLTQFLLHH**D**NARPHCSRATMTAIESLGFQVIPHPPYSPDLAPC**D**
FFLFPKLKEHLKGTKFNSDEKVKAEVKRWFNAQPEEFYLNGISKLVNRWQKCIALEGSYVEK

Apismar4.1

MERYSKEQRVLIVKTHYQNGEHYAVTVRKLRTILGHHNAPNESTVRRLIKKFEESGSTQDKKISGRHRSGRSEAN
VTVVHDSVTVSPRKSCRRRAQEMHMSPATMQRILTKDLHLHAYKVQ**LTQELK**PADHEKRRQFVEWILTRDRESEG
FAKRIIFT**D**EAHFHLNGFVNKQNCRIWGSENPRTIQEKEMHPERVTVWCGIWSGGLIGPYFFEDEEGNAVTVNGV
RYRAMLNHFLWPRLDQMNIENVWFQQ**D**GATCHTSRETIALLREKFPDTLISLRGDQSYPPRSCDLTPC**D**FFLWGY
TKSRVYQNKVRNVLELKQEIRCVLNELDGAMCDRVMVNFMERIIAYRASRGGHMPDVVFHC

Apismar4.2

gMNGYSVEQRVRIIKFYYQNQCSVRETFRAFTDFYPRHNRPAESTIRRLVAKF*STGSINYQPTPIRQRNARSIE
NIAAVRDSVRENPRQ*IPRRSQELGLSVTSTWRILRRDLGLHPYKIQ**LTQALK**VNDHTQRRVFADWVLGQLAVDP
NFAKKIIFS**D**EAHFWMNGYVNKQNCRIWDDTNPHKTHQNKMHPEEVTVWCGFWSGGIIGPYFFQNETGIAITVNG
ERYRSMINNFFWPKLDDMDTEDMWFQQ**D**GATCHTARATMDILRERFEGMVISRNGDINWPPRSCDLTPL**D**FFLWG
YLKSQVYANKPQTIDALKVNIINTIKKIQPDVCNKVIENWTTRIRATKQSRGGHLNDVIFHK

Apismar4.3

MVWTGVHRSFVVRAYYENNHSLIATQRAFRIHFGIPRNESIPSANTIKFWIRQLEETGSTLSELGHGAPRTVRTP
ENVQLVRESIEQSPTRSARKHAVALGISVRSLRRILHEYLSFHPYKLM**LVQELH**ATDYDNRKNLCQQILLRIPPT
STFFCS**D**EAHFHLSGTVNKQNFRYWAANNPQQLHERPLHSPKVTVWCGVSQFGVIGPYFFEDENRTVIVTPGRYV
VMLETYLQQRLEEMAEYHNLENVWFQQ**D**GATAHTAQISLGVLQQMFPGRLVSLRGDIGWPARSPDLSMC**D**FFLWG
YLKDKVFRHRPHTIEDLKQKITEEIEAIPVETCRKSYESFRDRLQQCIDADGRHHRDIISKQ

Apismar5.1

MLVVILVRLVGLNVKMNNQEKVQMLLIYGKCDRNSRQSARMYAEQYPGRYHPPHTFFIKIEQLLINHGAFSVKVV
RNQQIRENNINEDVELQVLAYIRLNPRSSVRHVGREVGISFGLVHKILKKHKMHPYKPD**LVQHLR**PADPERRLNF
IAWLLVQIDTKPLFLNQILWT**D**ESKFTNNGVINKQNNRMWSDVNPHWAVDNRYQTVWGTNVWCGLIGGKLLGPYF
YEENLTARRYLAFLTNVLPLMLENLPLATRQTLYFQQ**D**GAPAHNAHIVRDYLNRVYEGKWLGTYGPIEWPARSPD
ITPL**D**FFLWGHLKTVVYADPPVNLADLKNKILVACNNLTESQIMSATNRGCLQRFQLCVDNHGANFEQFI

Apismar5.2

MPSYSNTELPDMHFIYGLCNGNTRASQREYENRFPHRRVPAPAMFSRIHQALRQRGNFRRSLRESVQNVDLEREI
LDEVNRDPETSTRTLAHQFGVHHSTVWRTINREGLHPYHF*EFMA*RTQTINNVYSSVDGYFIMKLRIVVFSKVL
WT**D**ESTFTREGVFNIHNSHHYAQENPRLVRQQRFQRRFSINVWMGIIGGVLIGPFLGLPRTVGGNSYLNFLQNEL
PGLLEDLPLEVRRRMIYQH**D**GAPPHFSRAVRQHLDETFTCWIGRGGTIPWPPRSPDLTPM**D**FFVWGYLKERVYHQ
EVDSEAELRQRILQAAIEIRRVVTAGVTGRHVRERARACLRQNGGHIEQLL
