# Supplementary material for: Diversity and evolution of mariner-like elements in aphid genomes
Source: BMC Genomics. 2017 Jun 29;18:494. doi: 10.1186/s12864-017-3856-6 (PMC5490172; doi:10.1186/s12864-017-3856-6)
Supplement: Supplementary file 3 — Sequences classified by UPGM-VM method according to the reading sense indicated by the arrow in the circular tree. Deleted or truncated sequences are indicated by an asterisk (*). (PDF 276 kb) [file 12864_2017_3856_MOESM3_ESM.pdf]

Additional file 3. Sequences classified by UPGM-VM method according to the reading sense indicated by the arrow in the circular tree. Deleted or truncated sequences are indicated by an asterisk (*).

	Species	Accession numbers	Corresponding sequences
		***Macrosiphinimar* DD34D**	
1	*Diuraphis noxia*	gi984744883:140935-140721 strand-	*
2		gi984744980:13238-14654 strand+	*
3		gi984745316:3270-2865 strand-	*
4		gi984745505:879619-879165 strand-	*
5	*Acyrthosiphon pisum*	gi320446981 NW003383590.1 Scaffold101:202225-201649 strand -	*
6		gi320388812 NW003403215.1 Scaffold19726:243-904 strand -	*
7		gi320446984 NW003383587.1 Scaffold 98:39716-40668 strand +	MITE1.1 sub1
8	*Myzus persicae*	Scaffold419:92618-93582 strand-	*
9	*Acyrthosiphon pisum*	gi320446985 NW003383586.1 Scaffold97:172262-173595 strand +	Apismar1.1
10	*Myzus persicae*	Scaffold938 :36419-37755 strand-	Mpmar1.1
11		Scaffold42 :437239-438574 strand-	
12		Scaffold26 :74179-75510 strand+	
13		Scaffold353:292415-290211 strand -	
14		Scaffold167:418817-420155 strand+	
15		Scaffold425:148657-149615 strand +	MITE1.3
16	*Diuraphis noxia*	gi984745178:34523-35477 strand+	*
17	*Acyrthosiphon pisum*	gi320447035 NW003383536.1 Scaffold47:642566-643516 strand +	MITE1.1 sub1
18	*Myzus persicae*	Scaffold6:1582921..1583927 strand+	MITE1.3
19	*Acyrthosiphon pisum*	gi320446957 NW003383614.1 Scaffold125:833628-834589 strand -	MITE1.1 sub1
20	*Myzus persicae*	Scaffold344:286764-288093 strand+	Mpmar1.1
21		Scaffold50:754375-755343 strand+	*
22	*Acyrthosiphon pisum*	gi320446934 NW003383637.1 Scaffold 148:254344-255290 strand +	MITE1.1 sub1
23		gi320446899 NW003383672.1 Scaffold 183:345754-346918 strand +	
24	*Trachymyrmex cornetzi*	gi1006854094 LKEY01038001.1 Contig 38001:392-1605	
25	*Rhodnius prolixus*	Rpmar33 (Filée et *al.* 2015)	
26	*Vollenhovia emeryi*	gi763991541 BBUO01005411.1 Contig06950:10342-11676	
27	*Oncopeltus fasciatus*	gi641099433 JHQO01163363.1 ContigNC163363:6062-7386	
28	*Homalodisca vitripennis*	gi642862357 JJNS01041406.1 ContigNC41406:3310-4634	
29	*Myzus persicae*	Scaffold1103:23507-23906 strand+	*
30	*Diuraphis noxia*	gi984745202:272244-271837 strand-	*
31		gi984745488:452325-450979 strand-	Dnomar1.1
32		gi984745255:324638-325555 strand+	*
33	*Myzus persicae*	Scaffold131:48139-48677 strand-	*
34	*Acyrthosiphon pisum*	gi320447046 NW003383525.1 Scaffold36 :908811-909602 strand -	*
35		gi320447034 NW003383537.1 Scaffold48:638002-638846 strand +	*
36		gi320446875 NW003383696.1 Scaffold207:537540-538487 strand -	*
37	*Diuraphis noxia*	gi984745157:267924-268872 strand+	*
38		gi984744390:37892-38851 strand+	*
39	*Acyrthosiphon pisum*	gi320447089 NW003383510.1 Scaffold21:987841-988816 strand +	*
40		gi320446282 NW003384289.1 Scaffold800:146539-147106 strand -	MITE1.1 sub2
41		gi320446572 NW003383999.1 Scaffold510:194743-195684 strand +	
42		gi320446653 NW003383918.1 Scaffold429:453472-454426 strand +	
43		gi320446326 NW003384245.1 Scaffold756:48163-49727 strand +	
44		gi320447005 NW003383566.1 Scaffold77:451686-452577 strand +	*
45		gi320442452 NW003388040.1 Scaffold4551:1640-2495 strand +	*
46		gi320446993 NW003383578.1 Scaffold89:934560-935876 strand +	Apismar1.2
47	*Diuraphis noxia*	gi984745245:252450-251151 strand-	Dnomar1.2
48	*Dendroctonus ponderosae*	gi459605371 APGL01021548.1 Seq01021608:13322-14422	
49	*Gerris buenoi*	gi822390376 JHBY01085489.1 Contig85496: 109-1314	
50	*Homalodisca vitripennis*	gi642470880 JJNS01248885.1 ContigNC248885:1757-2877	
51	*Mesobuthus martensii*	gi553813549 AYEL01086727.1 Contig347321:2028-3230	
52	*Anoplophora glabripennis*	gi496870061 AQHT01063015.1 Contig63076:4332-5534	
		***Himar-like elements* DD34D**	

53	*Aphis glycines*	GQ231493	
54	*Drosophila yakuba*	Dromar18 (Wallau et *al*. 2014)	
55	*Rhodnius prolixus*	Rpmar0 (Filée et *al*. 2015)	
56	*Bactrocera dorsalis*	AF346541	
57	*Diachasmimorpha longicaudata*	AY601748	
58	*Chrysoperla plorabunda*	L06041	
59	*Haematobia irritans*	U11642	
60	*Mantispa pulchella*	U11649	
61	*Bactrocera dorsalis*	AY601743	
***Maibmar-like elements* DD34D**			
62	*Cancer pagurus*	AJ507245	
63	*Eriphia verrucosa*	AM906106	
64	*Thalamita possoinii*	AM906155	
65	*Pilumnus hirtellus*	AM906121	
66	*Xantho hydrophilus*	AM906156	
67	*Maia brachidactyla*	AJ507238	
***Bytmar-like element* DD34D**			
68	*Alvinella caudata*	AJ496120	
69	*Ventiella sulfuris*	AJ507232	
70	*Perisesarma bidens*	AM906146	
71	*Bythograea thermydron*	AJ507219	
72	*Portunus pelagicus*	AM906137	
73	*Alvinella pompejana*	AJ496135	
***Nosferatis* DD34D**			
74	*Rhodnius prolixus*	Rpmar13 (Filée et *al*. 2015)	
75		Rpmar9 (Filée et *al*. 2015)	
***Dnomar-like element* DD34D**			
76	*Diuraphis noxia*	gi984735891:2467-2015 strand-	*
77		gi984745098:54676-56035 strand+	Dnomar3.1
78	*Myzus persicae*	Scaffold280:15925-14979 strand -	*
79		Scaffold220: 401477-402088 strand +	*
***Hsmar2-like element* DD34D**			
80	*Portunus pelagicus*	AM906137	
81	*Alvinella pompejana*	AJ496135	
82	*Homo sapiens*	U49974	
83	*Lemur catta*	AC133072	
84	*Gorilla gorilla*	AC145402	
***Batmar-like element* DD34D**			
85	*Diuraphis noxia*	gi984745342:336477-336902 strand+	*
86		gi984744928:23881-24312 strand+	*
87		gi984745420:365693-366623 strand+	*
88		gi984745311:440129-439207 strand-	*
89	*Acyrthosiphon pisum*	gi320447037 NW003383534.1 Scaffold45:158750-158202 strand-	*
90		gi320446899 NW003383672.1 Scaffold183:261886-261320 strand-	*
91	*Diuraphis noxia*	gi984744320:45892-45397 strand-	*
92		gi984744320:47738-47243 strand-	*
93	*Acyrthosiphon pisum*	gi320446184 NW003384387.1 Scaffold898 :36060-36989 strand -	*
94		gi320446473 NW003384098.1 Scaffold609:132371-133021 strand+	*
95		gi320438813 NW003391455.1 Scaffold7966:4139-4600 strand+	*
96		gi320446392 NW003384179.1 Scaffold690:96269-97548 strand -	Apismar2.2
97		gi320447011 NW003383560.1 Scaffold71:493658-494941 strand +	
98		gi320447000 NW003383571.1 Scaffold82:64692-64037 strand -	*
99	*Heliconius melpomene*	gi378865014 CAEZ01008735.1 Contig7180001235928:24625-25792	
100	*Drosophila eugracilis*	gi449842783 AFPQ02005657.1 Contig5655:975265-976459	
101	*Neodiprion lecontei*	gi914279877 LGIB01001307.1 Scaffold1307:12116-13443	
102	*Lasius niger*	gi861599989 LBMM01019009.1:53-1374	
103	*Diuraphis noxia*	gi984745480:182128-183428 strand+	*
104		gi984745116:79279-80622 strand+	Dnomar2.2
105	*Agrilus planipennis*	gi648140536 JENH01008607.1 Contig8616:12243-13577	
106	*Diuraphis noxia*	gi984745529:295648-297002 strand+	Dnomar2.2

107	*Rhodnius prolixus*	Rpmar1 (Filée et *al.* 2015)	
108	*Dinoponera quadriceps*	gi938133368 JPHR01007292.1 Scaffold1145:2209123415	
109	*Copidosoma floridanum*	gi619889135 JBOX01069944.1 Contig69949:1149212815	
110	*Ceratitis capitata*	gi488305875 NW004523814.1 Contig13099:32902-34140	
111	*Drosophila ficusphila*	Dromar8 (Wallau et *al.* 2014)	
112	*Acyrthosiphon pisum*	gi320445628 NW003384943.1 Scaffold1454:8232- 8961 strand -	*
113		gi320445628 NW003384943.1 Scaffold1454:3454-4361 strand-	MITE2.1
114		gi320447015 NW003383556.1 Scaffold67:738385-739292 strand-	
115		gi320445203 NW003385294.1 Scaffold1805:8796-7866 strand-	
116		gi320446728 NW003383843.1 Scaffold354:173658-174753 strand -	*
117		gi320446952 NW003383619.1 Scaffold130:212179-213501 strand -	Apismar2.1
118	*Myzus persicae*	Scaffold544:202614-203522 strand+	MITE2.2
119		Scaffold166:292461-293374 strand+	
120		Scaffold6:1479795-1480706 strand+	
121	*Diuraphis noxia*	gi984744707:110647-111912 strand+	Dnomar2.1
122		gi984744972:124915-123590 strand-	
123	*Acyrthosiphon pisum*	gi320446088 NW003384483.1 Scaffold994:76058-76816 strand+	*
124	*Blattella germanica*	gi692674178 JPZV01249368.1 ContigNC249368:8211803	
125	*Trabutina mannipara*	gi1044319939 FKYK01006678.1 :1-1114	
126	*Camponotus floridanus*	gi304581076 AEAB01024585.1 Contig622:1601-2726	
127	*Trionymus perrisii*	gi1010807036 FIZV01000272.1 :504-1644	
128	*Trachymyrmex septentrionalis*	gi1006956206 LKEZ01022017.1 Contig22017:3294-4437	
129	*Homalodisca vitripennis*	gi642782498 JJNS01106073.1 ContigNC106073:3898-5039	
130	*Rhinolophus ferrumequinum*	AC157888	
131	*Wasmannia auropunctata*	gi780611046 XM011690486.1	
132	*Rhodnius prolixus*	Rpmar26 (Filée et *al.* 2015)	
133	*Carollia perspicillata*	AC148202	
***Pacmar-like element* DD34D**			
134	*Pachygrapsus marmoratus*	AM231069	
135		AM231072	
136	*Portunus granulatus*	AM906131	
137	*Pachygrapsus marmoratus*	AM983536	
138	*Portunus granulatus*	AM906134	
139		AM906132	
140	*Thalamita possoinii*	AM906151	
141	*Paromola bathyalis*	AM906119	
142	*Perisesarma bidens*	AM906150	
143	*Atelecyclus undecimdentatus*	AM906092	
***Other mariner elements* DD34D**			
144	*Papilio xuthus*	AB055185	
145	*Attacus atlas*	AB006464	
146	*Hyalophora cecropia*	M63844	
147	*Bombyx mori*	D88671	
148	*Antheraea yamamai*	AB247378	
149	*Antheraea mylitta*	AF126011	
150	*Homo sapiens*	EF517118	
151	*Apis mellifera*	AY155490	
152	*Forficula auricularia*	AY155492	
153	*Ceratitis capitata*	U76903	
154	*Caenorhabditis elegans*	U10438	
155	*Meloidogyne chiwoodi*	AJ437557	
156	*Caenorhabditis briggsae*	AC099767	
157	*Solenopsis invicta*	AF518170	
158	*Solenopsis saevissima*	AF518177	
159	*Myrmica ruginodis*	AY652423	
160	*Bombus terrestris*	AJ312712	
161	*Drosophila mauritiana*	M14653	
162		X78906	
163	*Drosophila simulans*	X89927	
164	*Mamestra brassicae*	AF465247	
165	*Messor bouvieri*	AJ781769	

166	*Mayetiola destructor*	gi30778846 AEGA01027875.1	
167		U24436	
168	*Rhynchosciara sp.*	GU442128	
***Crmar2-like element* DD41D**			
169	*Acyrthosiphon pisum*	gi320446057 NW003384514.1 Scaffold1025:1012-1344 strand+	*
170		gi320446057 NW003384514.1 Scaffold1025:2902-3244 strand+	*
171		gi320401144 NW003399120.1 Scaffold15631:349-1 strand-	MITE4.1 sub1
172		gi320447076 NW003383518.1 Scaffold29:1329370-1329000 strand-	
173		gi320446843 NW003383728.1 Scaffold239:535418-535797 strand+	
174		gi320446434 NW003384137.1 Scaffold648:49392-49775 strand-	
175		gi320446106 NW003384465.1 Scaffold976:179217-179591 strand+	
176		gi320446648 NW003383923.1 Scaffold434:173770-174148 strand+	
177		gi320446083 NW003384488.1 Scaffold999:5967-6345 strand+	
178		gi320447199 NW003383491.1 Scaffold2:2053341-2053729 strand+	
179		gi320447197 NW003383492.1 Scaffold3:641055-641464 strand -	*
180		gi320446904 NW003383667.1 Scaffold178:3884-4294 strand -	*
181		gi320441128 NW003389140.1 Scaffold5651:1032-1552 strand -	*
182		gi320447041 NW003383530.1 Scaffold41:488837-490191 strand -	Apismar4.1
183	*Diuraphis noxia*	gi984744291:33825-34544 strand+	*
184		gi984744522:27562-28176 strand+	*
185		gi984745358:548730-549307 strand+	MITE4.2
186		gi984745382:435077-434500 strand-	
187	*Acyrthosiphon pisum*	gi320447027 NW003383544.1 Scaffold55:451821-452338 strand+	*
188		gi320446929 NW003383642.1 Scaffold153:555302-554950 strand-	MITE4.1 sub2
189		gi320446959 NW003383612.1 Scaffold123:513503-513873 strand+	
190		gi320446812 NW003383759.1 Scaffold270:349975-349604 strand-	
191		gi320445944 NW003384627.1 Scaffold1138:18647-18271 strand-	
192	*Locusta migratoria*	AVCP010119604.1	
193	*Mesobuthus martensii*	gi553824729 AYEL01075810.1 Contig333646:1103812150	
194	*Stegodyphus mimosarum*	gi602493518 AZAQ01086514.1 Contig86514:26900-28235	
195	*Acyrthosiphon pisum*	gi320446734 NW003383837.1 Scaffold348:294674-294127 strand-	*
196		gi320445699 NW003384872.1 Scaffold1383:15032-14708 strand-	*
197		gi320447104 NW003383505.1 Scaffold16 :729100-731500 strand+	Apismar4.3
198	*Diuraphis noxia*	gi984745081:151016-151527 strand+	*
199		gi984745384:382222-382755 strand+	*
200		gi984745429:267344-266809 strand-	*
201		gi984745389:349961-349421 strand-	*
202		gi984745380:533248-533974 strand+	*
203		gi984745519:793804-794330 strand+	*
204		gi984745503:728303-727753 strand-	*
205	*Nilaparvata lugens*	gi688034042 AOSB01116314.1 Scaffold930:1140012616	
206	*Heliconius melpomene*	KU514436.1 GI:974707777 32163-33486	
207	*Acromyrmex echinatior*	XM011050935.1	
208	*Acyrthosiphon pisum*	gi320446279 NW003384292.1 Scaffold803:93845-94145 strand+	*
209		gi320445889 NW003384682.1 Scaffold1193:81099-81559 strand -	*
210		gi320446954 NW003383617.1 Scaffold128:616437-616899 strand-	*
211		gi320447044 NW003383527.1 Scaffold38:870577-871352 strand-	*
212		gi320446184 NW003384387.1 Scaffold898:33860-34319 strand+	*
213		gi320447097 NW003383508.1 Scaffold19:246121-246683 strand+	*
214		gi320446974 NW003383597.1 Scaffold108:252404-252965 strand-	*
215		gi320447085 NW003383512.1 Scaffold23:1752408-1753706 strand-	Apismar4.2
216		gi320447044 NW003383527.1 Scaffold38:871366-871812 strand+	*
217	*Atta cephalotes*	gi295962919 ADTU01003898.1 Contig03898:64257703	
218	*Trachymyrmex cornetzi*	gi1006767341 LKEY01058653.1 Contig58653:2926-4236	
219	*Atta colombica*	gi1006787365 LKEW01025605.1 Contig25605:1490716198	
220	*Danaus plexippus*	gi357604284 AGBW01012371.1 :21719-22917	
221	*Dufourea novaeangliae*	gi919891211 LGHO01003380.1 Contig3380:23632-24843	
222	*Harpegnathos saltator*	gi304616639 AEAC01015863.1 Contig6619:7536976586	
223	*Anoplophora glabripennis*	gi496927062 AQHT01044136.1 Contig44173:546-1789	
224	*Anastrepha suspensa*	AY034629	
225		AY034630	
226	*Operophtera brumata*	gi914552887 JTDY01008752.1 2357-3647	

227	*Ceratitis rosa*	AY034623	
228	*Homalodisca vitripennis*	gi642494493 JJNS01240973.1 ContigNC240973:3989-5223	
229	*Anoplophora glabripennis*	gi496912294 AQHT01049420.1 Contig49462:67688047	
230	*Dendroctonus ponderosae*	gi459588353 APGL01038566.1	
231	*Acyrthosiphon pisum*	gi320446058 NW003384513.1 Scaffold1024:28892-29972 strand-	*
232		gi320446789 NW003383782.1 Scaffold293:337514-338606 strand +	*
233	*Metaseiulus occidentalis*	gi391326941 XM003737920.1	
***LTIR-like element* DD40-41D**			
234		gi320446742 NW003383829.1 Scaffold340:1803-2145 strand+	*
235		gi320446952 NW003383619.1 Scaffold130:17385-18186 strand+	*
236		gi320442315 NW003388177.1 Scaffold4688:7007-6080 strand-	*
237		gi320446920 NW003383651.1 Scaffold162:358972-359636 strand+	*
238		gi320447020 NW003383551.1 Scaffold62:504081-503547 strand-	*
239		gi320447044 NW003383527.1 Scaffold38:494414-493847 strand-	*
240		gi320447038 NW003383533.1 Scaffold44:245172-244580 strand-	*
241	*Acyrthosiphon pisum*	gi320442393 NW003388099.1 Scaffold4610:542-1295 strand+	*
242		gi320433456 NW003396117.1 Scaffold12628:816-1 strand-	*
243		gi320446328 NW003384243.1 Scaffold754:22810-24151 strand+	*
244		gi320446852 NW003383719.1 Scaffold230:461388-461756 strand+	*
245		gi320447106 NW003383503.1 Scaffold14:910161-909675 strand-	*
246		gi320447031 NW003383540.1 Scaffold51:481283-479084 strand-	*
247		gi320446462 NW003384109.1 Scaffold620:170760-168538 strand-	*
248		gi320447015 NW003383556.1 Scaffold67:301784-299478 strand-	Apismar5.1
249		Scaffold228: 352709..353122 strand+	*
250	*Myzus persicae*	Scaffold16:1450072-1450945 strand+	*
251		Scaffold176:395279-395722 strand+	*
252		Scaffold10:1542469-1543323 strand-	*
253		gi984745065:239246-238443 strand-	
254		gi984745517:765990-765175 strand-	MITE5.1
255		gi984745390:259040-259829 strand+	
256	*Diuraphis noxia*	gi984745032:193491-194325 strand+	*
257		gi984745175:253583-254179 strand+	*
258		gi984745400:243420-244241 strand+	MITE5.1
259		gi984745050:86317-85518 strand-	
260		gi984745081:249492-250419 strand+	*
261		gi320446972 NW003383599.1 Scaffold110:354919-355343 strand+	*
262		gi320447020 NW003383551.1 Scaffold62:503547-504119 strand+	*
263	*Acyrthosiphon pisum*	gi320446800 NW003383771.1 Scaffold282:207301-208570 strand+	*
264		gi320446154 NW003384417.1 Scaffold928:21356-19996 strand-	*
265		gi320446987 NW003383584.1 Scaffold95:302694-301750 strand-	*
266		gi320447105 NW003383504.1 Scaffold15:1038009-1036576 strand-	*
267	*Diuraphis noxia*	gi984745508:982719-984070 strand+	*
268		gi320447008 NW003383563.1 Scaffold74:94584-96017 strand+	*
269		gi320442315 NW003388177.1 Scaffold4688:6080-7007 strand+	*
270		gi320446319 NW003384252.1 Scaffold763:18048-18455 strand+	*
271	*Acyrthosiphon pisum*	gi320447082 NW003383514.1 Scaffold25:979065-978002 strand-	*
272		gi320446163 NW003384408.1 Scaffold919:2730-3812 strand-	*
273		gi320447077 NW003383517.1 Scaffold28:658930-658046 strand-	*
274		gi320447191 NW003383495.1 Scaffold6:867979-866906 strand-	*
275	*Myzus persicae*	Scaffold1040:29624-30705 strand+	*
276		Scaffold1981:5634-7149 strand+	*
277		gi320446879 NW003383692.1 Scaffold203:311633-311055 strand-	*
278	*Acyrthosiphon pisum*	gi320447177 NW003383497.1 Scaffold8:947336-948127 strand+	*
279		gi320446579 NW003383992.1 Scaffold503:316494-315441 strand-	*
280	*Diuraphis noxia*	gi984745469:593111-591544 strand-	*
281		gi320447082 NW003383514.1 Scaffold25:163596-162726 strand-	*
282	*Acyrthosiphon pisum*	gi320434094 NW003395907.1 Scaffold12418:13-883 strand+	*
283		gi320392825 NW003401900.1 Scaffold18411:6357-5063 strand-	*
284	*Bombus terrestris*	gi339751187 AELG01000709: 6387911-6385650	
285	*Nasonia vitripennis*	gi154053393:26728-27797	
286	*Harpegnathos saltator*	NW011646526.1 Scaffold1231:24830-28107	
287	*Pogonomyrmex barbatus*	NW011933482.1 Scaffold7180000350099:45895-49565	

288	*Calycopis cecrops*	LUGF01035226.1	
289	*Cimex lectularius*	JRLE01000220.1 Scaffold310843	
290	*Rhodnius prolixus*	KQ034065 Scaffold9:2936024-2933697	
291	*Anoplophora glabripennis*	gi496979286 AQHT01025496.1 Contig25506:11686-12762	
292	*Homalodisca vitripennis*	KK962044.1 Scaffold551:1019188-1021387	
293	*Megachile rotundata*	gi383080544 Scaffold0130:560690-562464	
294	*Acyrthosiphon pisum*	gi320447045 NW003383526.1 Scaffold37:902476-902066 strand+	MITE5.2 sub1
295		gi320446511 NW003384060.1 Scaffold571:438535-438949 strand+	
296		gi320446471 NW003384100.1 Scaffold611:61351-61779 strand+	
297		gi320446667 NW003383904.1 Scaffold415:160458-160887 strand+	
298		gi320446129 NW003384442.1 Scaffold953:162133-162549 strand+	MITE5.2 sub2
299		gi320446884 NW003383687.1 Scaffold198:591126-591545 strand+	
300		gi320446815 NW003383756.1 Scaffold267:153990-154430 strand+	
301		gi320447083 NW003383513.1 Scaffold24:949317-949750 strand+	*
302		gi320447001 NW003383570.1 Scaffold81:754854-757276 strand+	Apismar5.2
303		gi320446306 NW003384265.1 Scaffold776:57772-58831 strand+	*
304		gi320446984 NW003383587.1 Scaffold98:28691-29172 strand+	*
305		gi320446449 NW003384122.1 Scaffold633:131583-130926 strand-	*
306		gi320446999 NW003383572.1 Scaffold83:210237-208812 strand-	*
307		gi320446763 NW003383808.1 Scaffold319:416807-419008 strand+	*
***maT* DD37D**			
308	*Caenorhabditis elegans*	AF038612	
309		Z83129	
310	*Caenorhabditis briggsae*	AC084524	
311	*Bombyx mori*	U43131	
312	*Anopheles gambiae*	AAAB01008975	
Outgroup TLE and IS630			
313	*Fusarium oxysporum*	AF282722	
314	*Caenorhabditis briggsae*	M64308	
315	*Drosophila virilis*	CH940657	
316	*Salmo salar*	AJ249090	
317	*Anopheles gambiae*	U89802	
318	*Aedes aegypti*	AF208675	
319	*Caenorhabditis elegans*	M77697	
320	*Aedes atropalpus*	AY038027	
321	*Fusarium oxysporum*	AF076632	
322	*Fusarium solani*	AF443562	
323	*Fusarium oxysporum*	AF076631	
324		AJ608703	
325	*Aspergillus niger*	U58946	
326	*Sinorhizobium meliloti*	AF143444	
327	*Pseudomonas sp.*	U15298.1	
328	*Catharanthus roseus*	DQ852611	
329	*Salmonella typhimurium*	M58505	
