# Supplementary material for: Diversity and evolution of mariner-like elements in aphid genomes
Source: BMC Genomics. 2017 Jun 29;18:494. doi: 10.1186/s12864-017-3856-6 (PMC5490172; doi:10.1186/s12864-017-3856-6)

Additional file 4. TIR sequences for each clade. TIR consensus per clade was generated using the Web-Logo server (http://weblogo.berkeley.edu/logo.cgi). At each position the nucleotides are stacked one on top of another with the most frequent one on the top. It displays the frequency of bases at each position with height indicating the proportion of occurrence. The vertical scale is in bits with maximum of two bits possible at each position indicating that there can be possibility of four different bases at each position. For LTIR- like element, only the first 57 nucleotides are presented.

Consensus of TIR-*irritans*

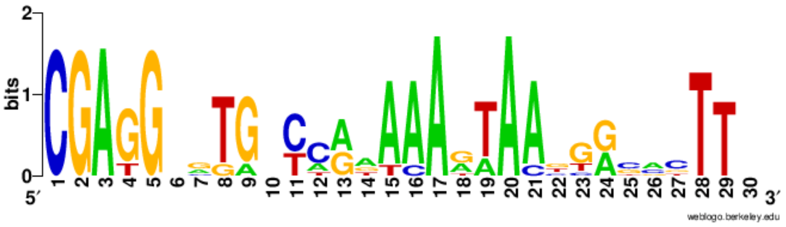

Consensus of TIR-*rosa*

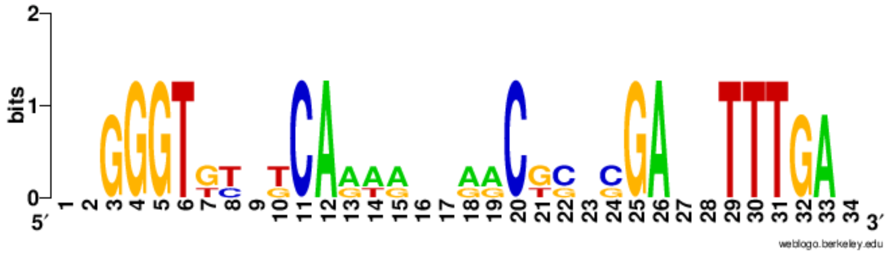

Consensus of TIR-*LTIR*

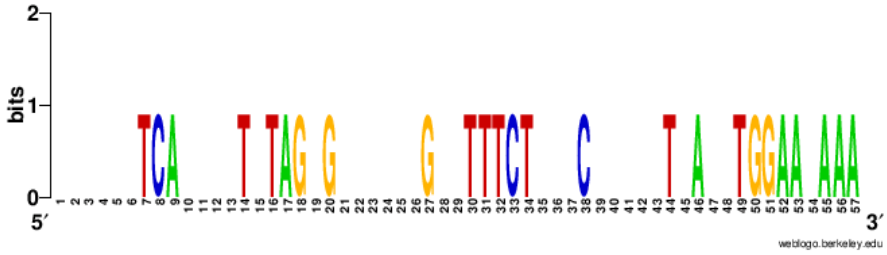

Supplement: Supplementary file 4 — TIR sequences for each clade. TIR consensus per clade was generated using the Web-Logo server (http://weblogo.berkeley.edu/logo.cgi). At each position the nucleotides are stacked one on top of another with the most frequent one on the top. It displays the frequency of bases at each position, with height indicating the proportion of occurrence. The vertical scale is in bits with maximum of two bits possible at each position, indicating that there can be possibility of four different bases at each position. For LTIR- like elements, only the first 57 nucleotides are presented. (PDF 111 kb) [file 12864_2017_3856_MOESM4_ESM.pdf]
