# Supplementary material for: Diversity and evolution of mariner-like elements in aphid genomes
Source: BMC Genomics. 2017 Jun 29;18:494. doi: 10.1186/s12864-017-3856-6 (PMC5490172; doi:10.1186/s12864-017-3856-6)
Supplement: Supplementary file 5 — Pairwise divergence matrix between amino acid lineages. Fifteen complete sequences have been aligned using Aliview, The alignment was then transferred in GENEDOC software [65] to obtain the identity percentage. Sequences name: Apismar: elements from Acyrthosiphon pisum, Dnomar: elements from Diuraphis noxia and Mpmar: elements from Myzus persicae. (PDF 354 kb) [file 12864_2017_3856_MOESM5_ESM.pdf]

Additional file 5. Pairwise divergence matrix between amino acid lineages. Fifteen complete sequences have been aligned using Aliview. The alignment was then transferred in GENEDOC software [64] to obtain the identity percentage.

Sequences name: *Apismar*: elements from *Acyrthosiphon pisum*, *Dnomar:* elements from *Diuraphis noxia* and *Mpmar*: elements from *Myzus persicae.*

	Macrosiphinimar					***Batmar-like element***				***Dnomar-like element***	***Rosa***			***LTIR-like element***	
	Apismar1.1	Apismar1.2	Dnomar1.1	Dnomar1.2	Mpmar1.1	Apismar2.1	Apismar2.2	Dnomar2.1	Dnomar2.2	Dnomar3.1	Apismar4.1	Apismar4.2	Apismar4.3	Apismar5.1	Apismar5.2
Apismar1.1		66%	88%	62%	95%	54%	49%	47%	52%	58%	32%	31%	30%	29%	33%
Apismar1.2			62%	79%	67%	50%	48%	43%	48%	54%	34%	32%	31%	32%	34%
Dnomar1.1				59%	89%	53%	48%	46%	52%	55%	32%	32%	29%	28%	33%
Dnomar1.2					63%	49%	45%	42%	43%	52%	32%	33%	30%	31%	34%
Mpmar1.1						55%	51%	47%	52%	59%	33%	33%	31%	30%	33%
Apismar2.1							66%	78%	64%	53%	36%	37%	36%	34%	34%
Apismar2.2								56%	66%	50%	35%	35%	37%	31%	32%
Dnomar2.1									53%	44%	32%	34%	33%	34%	31%
Dnomar2.2										52%	35%	34%	35%	32%	32%
Dnomar3.1											36%	32%	38%	35%	33%
Apismar4.1												68%	60%	44%	42%
Apismar4.2													56%	46%	44%
Apismar4.3														43%	41%
Apismar5.1															52%
Apismar5.2															
